# The impact of support from emergency nurse organizations on compassion fatigue: the mediating role of psychological capital

**DOI:** 10.3389/fpubh.2025.1551381

**Published:** 2025-04-25

**Authors:** Daixun Xie, Xiaoqin Zhu, Xiurong Zhang, Zhaoquan Jiang, Tao Liu

**Affiliations:** Emergency Department, Yibin Second People's Hospital, Yibin, China

**Keywords:** emergency nurse, compassion fatigue, psychological capital, organizational support, mediating effect

## Abstract

**Background:**

With ongoing healthcare system reforms in China and escalating demands for emergency services, emergency nurses frequently face high-pressure, complex work environments. This increases their psychological burden. Additionally, limited healthcare resources at the grassroots level and high workloads heighten their vulnerability to compassion fatigue. Current research into the interplay between psychological capital, perceived organizational support, and compassion fatigue among emergency nurses is limited, particularly regarding the mediating role of psychological capital.

**Objective:**

This study aims to explore the relationship between organizational support, psychological capital, and compassion fatigue among emergency nurses. It specifically investigates the mediating role of psychological capital in the relationship between organizational support and compassion fatigue.

**Methods:**

A total of 466 emergency nurses from medical institutions in the Chengdu-Chongqing area were selected through convenience sampling. Data were collected using the Psychological Capital Questionnaire, the Sense of Organizational Support Scale, and the Compassion Fatigue Scale. The correlations among these variables were analyzed using SPSS 26.0, and the mediating effects were tested using AMOS 26.0.

**Results:**

There is a significant negative correlation between psychological capital and compassion fatigue among emergency nurses (*r* = −0.309, *P* < 0.01), as well as between organizational support and compassion fatigue (*r* = −0.449, *P* < 0.01). Psychological capital acts as a partial mediator in the relationship between organizational support and compassion fatigue, with a mediating effect value of −0.169, accounting for 27% of the total effect.

**Conclusion:**

The findings indicate a significant association between psychological capital, perceived organizational support, and compassion fatigue among emergency nurses, with psychological capital playing a mediating role. Enhancing psychological capital and perceived organizational support for emergency nurses is an effective strategy for managing emotional stress at work. Healthcare institutions should implement targeted interventions to improve nursing service quality and promote the sustainable development of the nursing profession.

## 1 Introduction

The frequency of public health events across the globe, coupled with ongoing pressures on healthcare resources, places nurses at the forefront of the healthcare system. As vital caregivers, particularly in high-risk environments such as emergency and critical care, nurses are directly engaged with patient illnesses and are pivotal in providing care. Research has indicated that emergency department nurses experience significantly higher rates of burnout compared to their counterparts in other nursing roles (1). A cross-sectional study of burnout among 179 HCW in the emergency department in Pakistan showed that 42.4% of the patients showed emotional fatigue [27A study of 19 HCWS conducted during the second wave of COVID-19 87 found that 54% of participants experienced psychological fatigue (2). Wijdenes et al. found in the current study of 315 nurses' compassion fatigue that about 46% of nurses had moderate to severe risk of compassion fatigue (3). The chronic occupational stress associated with handling traumatic events, along with the necessity to interact with patients and their families, often results in “empathy fatigue” (4). This condition not only diminishes nurses' productivity but also heightens the risk of medical errors.

Empathy fatigue is characterized by the indirect psychological trauma that medical staff endure due to empathetic responses during patient care, leading to clinical job burnout and a diminished capacity to empathize with patients (5, 6). Prior studies have identified the primary contributors to empathy fatigue among nurses as the empathetic responses elicited and the indirect exposure to traumatic stressors (7–9). In their roles, emergency nurses frequently provide immediate first aid in situations of disaster rescue, major traffic incidents, poisoning, burns, and more. Consequently, their indirect exposure to traumatic events is inevitable, which contributes to the development of empathy fatigue (10–12). Psychological capital refers to the positive psychological state exhibited by individuals during their growth and development. It is a core psychological element that complements human and social capital and serves as a psychological resource to foster growth and enhance the performance of nurses (13, 14). The concept of organizational support refers to employees' perceptions of the positive backing they receive from their organization. This support leads to a positive evaluation of the organization, prompting employees to respond and provide feedback positively and to strive toward achieving the organization's goals (15–18).

A cross-sectional study conducted by Liu in 2024 demonstrated a significant negative correlation between nurses' perceptions of organizational support and empathy fatigue (19). Furthermore, a study by Kong et al. confirmed that psychological capital acts as a partial mediator in the relationship between organizational support and the career engagement of nursing faculty (20). This finding highlights the significant role of psychological capital in the career development of nursing educators, suggesting that it can be an intrinsic resource for coping with work challenges, maintaining enthusiasm, and enhancing professional competence. This notion is supported by research conducted by Ma and Zheng et al., which identified organizational support as a crucial factor in boosting nurses' wellbeing and job performance (21, 22). However, most prior studies have directly examined the relationship between organizational support and nurses' wellbeing or job performance, primarily focusing on the educational or business sectors. Nevertheless, the role of psychological capital as a mediator between organizational support and empathy fatigue, particularly in the field of emergency nursing, remains unverified (23–27). Based on this perspective, this study aims to elucidate the mediating role of psychological capital between perceived organizational support and empathy fatigue, providing a theoretical foundation for the development of future intervention strategies targeting empathy fatigue among emergency nurses.

The research hypotheses for this study are as follows:

Hypothesis 1: There is a negative correlation between emergency nurses' perception of organizational support and their experience of empathy fatigue.

Hypothesis 2: Psychological capital among emergency nurses is negatively associated with empathy fatigue.

Hypothesis 3: There is a positive relationship between the sense of organizational support and psychological capital among emergency nurses.

Hypothesis 4: Psychological capital mediates the relationship between perceptions of organizational support and empathy fatigue among emergency nurses.

## 2 Objects and methods

### 2.1 Participants and procedures

This survey uses the questionnaire made by the questionnaire star. We contacted nursing departments in various hospitals to obtain endorsement of the principles and objectives of the study, and trained instructors guided survey participants to carefully complete the questionnaire. From September to November 2024, emergency nurses from medical institutions in the Yicheng and Chongqing areas were randomly selected as participants for the research. Inclusion criteria included: (1) nurses who have obtained a nursing practice certificate and signed a labor contract; (2) those engaged in emergency care for at least 1 year; and (3) those who voluntarily participated and were informed of the study's content and purpose. Exclusion criteria were: (1) nurses in rotation, training, or practice positions not yet confirmed as permanent staff; (2) nurses in nursing management positions; (3) nurses unable to understand the content of the assessment tool; (4) nurses with a mental disorder. The study utilized the Beck Depression Inventory, Second Edition (BDI-2), translated and validated by Chinese scholar Wang Zhen (28). Nurses scoring at a critical level (13) on these measures were excluded from the study.

### 2.2 Sample size calculation

Based on Kendell's rough estimation method for sample size calculation (29), it is generally required that the sample size be at least 5 to 10 times the number of variables studied. In this study, there were 8 demographic variables, 20 psychological capital variables, 13 organizational support scale variables, and 13 empathy fatigue scale variables, totaling 54 variables. Therefore, the estimated required sample size ranged from 270 to 540 cases. Allowing for a 20% rate of invalid questionnaires, the final sample size was adjusted to between 324 and 648 cases. In this survey, 480 questionnaires were collected, of which 14 were deemed invalid, leaving 466 for final statistical analysis. The effective response rate was 97%.

### 2.3 Survey tools

#### 2.3.1 General demographic questionnaire

Drawing on relevant literature (19–22), researchers designed a General Demographic Questionnaire to gather demographic information from emergency nurses, including gender, age, years of employment, monthly income, marital status, childbearing status, number of night shifts, and health status.

#### 2.3.2 Organizational support scale

The perceived organizational support scale, as modified from previous research, was utilized. It comprises two dimensions: emotional support and instrumental support, with a total of 13 items (30). Scoring was conducted using a 5-level Likert scale, ranging from 1 (“extremely inconsistent”) to 5 (“extremely consistent”), with the total scoring range being 13 to 65 points. A higher score indicates stronger perceived organizational support. The Cronbach's Alpha value for this scale in the study was 0.848.

#### 2.3.3 Psychological capital questionnaire (PCQ-R)

We adopted a Chinese version of the revised Nurses' Psychological Capital Scale (31), which comprises 20 items across four dimensions. These include six items each in the self-efficacy and hope dimensions, five items in the resilience dimension, and three items in the optimism dimension. The scale utilizes a six-level Likert scoring method, ranging from 1 (“strongly disagree”) to 6 (“strongly agree”). The total score can range from 20 to 120 points, where a higher score indicates a greater level of psychological capital. The Cronbach's Alpha value for the total scale in this study was 0.884.

#### 2.3.4 The Chinese version of the empathy fatigue brief scale (CF)

The revised Chinese version of the Empathy Fatigue Brief Scale was used (32), which includes two dimensions and 13 items. Of these, secondary trauma comprises five items, and job burnout comprises eight items. Each item is scored using a 10-level Likert method, from “never” to “very frequent,” with scores ranging from 1 to 10. The total score varies from 13 to 130 points, with higher scores indicating a more severe perception of empathy fatigue. The Cronbach's Alpha value for the total scale in this study was 0.893.

### 2.4 Statistical methods

Data analysis was conducted using SPSS 26.0 software. Count data were presented through frequency and percentage, while normally distributed measurement data were expressed as mean ± standard deviation (x ± s). Pearson correlation analysis explored the relationships between empathy fatigue, organizational support, and psychological capital. Multiple linear regression models were developed to assess the impact of perceived organizational support and psychological capital on empathy fatigue, controlling for potential confounders such as gender. Structural equation modeling was constructed using AMOS 24.0, and the significance of mediation effects was tested using the Bootstrap method at a significance level of α = 0.05.

## 3 Results

### 3.1 General demographic data of emergency nurses

The study included 466 emergency nurses: 33 males (7.0%) and 433 females (92.9%). Age distribution was as follows: 239 individuals (51.3%) aged 20–30, 192 (41.2%) aged 31–40, and 35 (7.5%) aged 40 or older. Regarding marital status, 286 nurses were married (61.4%), 184 were unmarried (39.5%), and 14 were divorced (3.0%). Differences in empathy fatigue were observed in relation to gender, number of night shifts, and health status, as detailed in Table 1.

**Table 1:** Univariate analysis of empathy fatigue of emergency nurse organizations with different characteristics (*N* = 466).

**Variable**	**Grouping**	**Frequency**	**Percent (%)**	**Mean ±SD**	***F/t***	***P***
Gender	Male	33	7.08	67.364 ± 22.960	5.781	0.017
	Female	433	92.92	59.340 ± 18.105		
Age	20–30 years old	239	51.29	60.130 ± 18.827	0.072	0.931
	31–40 years old	192	41.20	59.531 ± 18.227		
	≥40 years old	35	7.51	60.457 ± 19.233		
Marriage	Unmarried	184	39.48	61.337 ± 19.111	1.023	0.360
	Married	268	57.51	59.101 ± 18.382		
	Get divorced	14	3.00	56.571 ± 14.373		
Fertility status	Be childless	206	44.21	61.097 ± 18.745	0.944	0.390
	1 Child	157	33.69	58.395 ± 19.404		
	2 Children	103	22.10	59.835 ± 16.869		
Length of service	1–5 years	187	40.13	60.032 ± 19.182	0.944	0.390
	5–10 years	78	16.74	63.167 ± 20.220		
	≥10 years	201	43.13	58.527 ± 17.220		
Number of night shifts	No night shift	69	14.81	57.362 ± 19.317	5.794	0.003
	1 5	206	44.21	63.165 ± 19.492		
	6 10	191	40.99	57.314 ± 16.743		
Monthly income	< 5,000 yuan	190	40.77	61.605 ± 18.766	1.342	0.262
	5,000 7,999 yuan	227	48.71	58.727 ± 18.356		
	≥8,000 yuan	49	10.52	58.796 ± 18.715		
State of health	Good	198	42.49	55.010 ± 18.126	13.566	0.001
	Medium	237	50.86	62.975 ± 18.451		
	Worse off	31	6.65	67.742 ± 14.713		

### 3.2 Common method bias test

Exploratory factor analysis of the questionnaire items was conducted using Harman's single-factor test. Post-rotation, nine factors with eigenvalues over 1 were identified, accounting for a cumulative variance of 68.59%. The variance extracted by the first factor was 32.61%, which is below the critical criterion of 40%, indicating that common method bias is not present (33, 34).

### 3.3 Empathy fatigue, sense of organizational support, and psychological capital scores among emergency nurses

This study involved 466 emergency nurses who reported scores for empathy fatigue (94.43 ± 22.87), perceived organizational support (41.54 ± 12.35), and psychological capital (77.89 ± 10.73), as detailed in Table 2.

**Table 2:** Scores of empathy fatigue, sense of organizational support and psychological capital of emergency nurses (*n* = 466, x̄ ± s).

**Dimensionality**	**Score**	**Item mean score**
Sense of organizational support	41.54 ± 12.35	3.01 ± 0.84
Emotional support	29.23 ± 9.63	3.02 ± 0.87
Instrumental support	12.30 ± 2.71	3.10 ± 0.85
Empathy fatigue	94.43 ± 22.87	7.64 ± 0.50
Secondary trauma	37.39 ± 16.99	6.47 ± 0.57
Job burnout	56.50 ± 18.74	7.81 ± 0.51
Psychological capital	77.89 ± 10.73	3.82 ± 1.01
Self-efficacy	22.42 ± 8.68	3.90 ± 0.99
Hope for	17.42 ± 5.95	3.73 ± 1.05
Toughness	18.12 ± 6.67	3.82 ± 1.04
Optimism	20.45 ± 6.56	3.81 ± 1.14

### 3.4 Analysis of the relationship between empathy, organizational support, and psychological capital in emergency nurses

There was a significant negative correlation between empathy fatigue and organizational support among emergency nurses (*r* = −0.449, *P* < 0.01). Similarly, a significant negative correlation existed between empathy fatigue and psychological capital among these nurses (*r* = −0.309, *P* < 0.01), as illustrated in Table 3.

**Table 3:** Correlation analysis of empathy fatigue, sense of organizational support and psychological capital among emergency nurses (*n* = 466).

**Dimensionality**	**Empathy fatigue**	**Secondary trauma**	**Job burnout**
Sense of organizational support	−0.449^**^	−0.397^**^	−0.486^**^
Emotional support	−0.451^**^	−0.398^**^	−0.489^**^
Instrumental support	−0.423^**^	−0.375^**^	−0.457^**^
Psychological capital	−0.309^**^	−0.253^**^	−0.355^**^
Self-efficacy	−0.248^**^	−0.200^**^	−0.288^**^
Hope for	−0.294^**^	−0.240^**^	−0.339^**^
Toughness	−0.296^**^	−0.243^**^	−0.340^**^
Optimism	−0.335^**^	−0.278^**^	−0.382^**^

^**^p < 0.01.

### 3.5 Linear regression analysis

Multiple linear regression analyses were performed three times, with empathy fatigue as the dependent variable, sociodemographic characteristics as control variables, and organizational support and psychological capital as the main independent variables (refer to Table 4). According to Model 1, factors such as gender, number of night shifts, and health status significantly influenced empathy fatigue, with these general variables explaining 7.1% of the variance (*F* = 12.914, *P* < 0.001). Model 2 revealed that organizational support was significantly and negatively associated with empathy fatigue, accounting for 10.4% of the standardized variance (*F* = 14.535, Δ*R*^2^ = 0.104, *P* < 0.001). In Model 3, after adding psychological capital to Model 2, both the Organizational Support and Psychological Capital scales showed significant negative correlations with empathy fatigue, explaining 24.4% of the standardized variance (*F* = 30.951, ΔR^2^ = 0.244, *P* < 0.001). This indicates that psychological capital mediated the relationship between organizational support and empathy fatigue, as demonstrated in Table 4.

**Table 4:** Linear regression analysis results.

**Predictors**	**Model 1**				**Model 2**				**Model 3**			
	β	***SE***	***t***	**95%CI**	β	***SE***	***t***	**95%CI**	β	***SE***	***t***	**95%CI**
Gender	−0.125	3.242	−2.784^**^	−15.394, −2.653	−0.110	3.194	−2.487^*^	−14.221, −1.668	−0.094	2.938	−2.321^*^	−12.592, −1.047
Number of night shifts	−0.098	1.201	−2.154^*^	−4.947, −0.227	−0.101	1.180	−2.272^*^	−4.998, −0.362	−0.117	1.085	−2.853^**^	−5.228, −0.963
State of health	0.251	1.392	5.547^**^	4.988, 10.460	0.245	1.368	5.521^**^	4.865, 10.242	0.172	1.281	4.126^**^	2.767, 7.801
Sense of organizational support					−0.187	0.163	−4.240^**^	−1.012, −0.371	−0.092	0.154	−2.212^*^	−0.645, −0.038
Psychological capital									−0.394	0.053	−9.269^**^	−0.594, −0.386
*ΔR^2^*			0.071				0.104				0.244	
*F*			12.914				14.535				30.951	

^*^p < 0.05, ^**^p < 0.01.

### 3.6 Mediating effect of psychological capital on organizational support and empathy fatigue among emergency nurses

**Figure 1:**
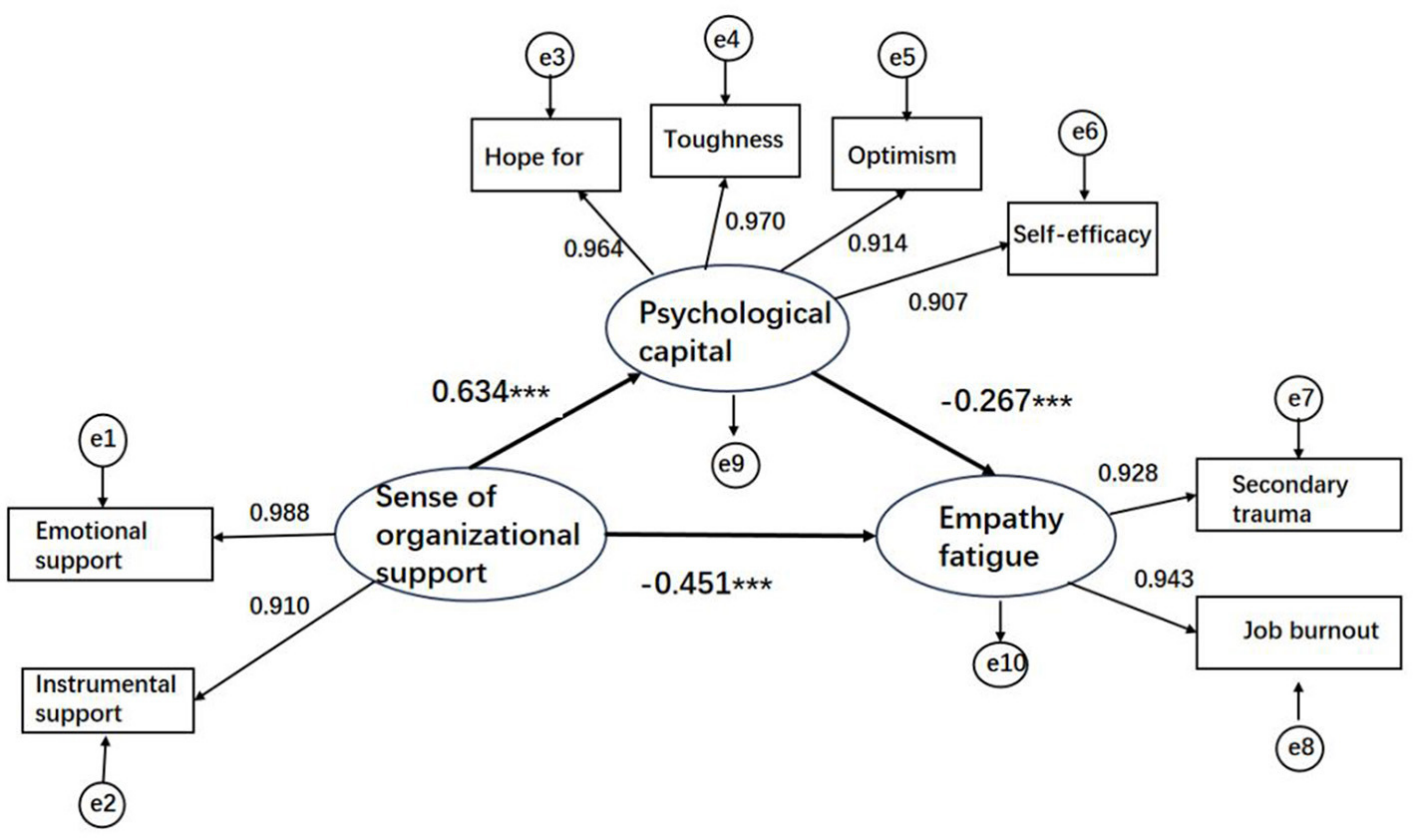
Structural equation model (standardized) of the relationship between psychological capital, organizational support and empathy fatigue among emergency nurses and nursing students. ***p < 0.001.

Based on the results of the correlation analysis, a hypothesis model was formulated with empathy fatigue as the dependent variable, organizational support as the independent variable, and psychological capital as the mediating variable (see Figure 1). All fit indices met the established standards, confirming a good model fit, as reported in Table 5. The Bootstrap method was applied to evaluate the significance of the mediating effect of psychological capital. The results indicated that the 95% confidence interval for the mediating effect of psychological capital between organizational support and empathy fatigue ranged from −0.216 to −0.112, excluding zero, thus confirming the significance of the mediating effect. The calculated mediating effect was 0.634 × −0.267 = −0.169, representing 27% of the total effect, as shown in Table 6.

**Table 5:** Indicators of overall goodness of fit of the model.

**Index of fit**	**TLI**	**AGFI**	**IFI**	**The chi-square degrees of freedom ratio χ2/df**	**GFI**	**RMSEA**	**RMR**
Judging standard	>0.9	>0.9	>0.9	< 3	>0.9	< 0.10	< 0.05
Value	0.960	0.853	0.976	2.786	0.930	0.121	0.078
**Index of fit**	**PGFI**	**PNFI**	**PCFI**	**SRMR**	**CFI**	**NFI**	**NNFI**
Judging standard	>0.5	>0.5	>0.5	< 0.1	>0.9	>0.9	>0.9
Value	0.539	0.590	0.592	0.032	0.975	0.972	0.960

**Table 6:** Results of Bootstarp mediation effect test.

**Parameters**	**Estimates of value**	**Lower limit**	**Upper limit**	**P**	**Effect proportion/%**
Indirect effect	−0.169	−0.216	−0.112	0.000	27
Direct effect	−0.451	−0.645	−0.476	0.000	73
Total effect	−0.620	−0.874	−0.634	0.000	100

## 4 Discussion

### 4.1 The situation of empathy fatigue and related differences

In this research, the mean empathy fatigue score among emergency nurses was recorded at 94.43 ± 22.87, which is consistent with previous research examining empathy fatigue within the domain of emergency nursing (25). The study also uncovered statistically significant variations in empathy fatigue scores among different demographic groups within these organizations, specifically related to gender, the frequency of night shifts, and overall health status. The findings indicate that female emergency nurses, those who worked fewer night shifts, and those reporting better health status demonstrated lower empathy fatigue scores. These results are in agreement with the conclusions drawn by McMillin (17). The societal inclination to ascribe more nurturing roles to women may contribute to female nurses adopting a more patient and meticulous approach in patient interactions. This societal expectation might also lead to stronger bonds of trust and reliance between female nurses and their patients and their families, fostering closer working relationships that can potentially alleviate empathy fatigue. In contrast, frequent night shifts place nurses under increased work pressure, as they are more likely to handle emergencies or critical cases during these hours. Reducing the frequency of night shifts could diminish this strain, affording nurses greater opportunities for psychological recuperation and stress relief (13). Moreover, the health status of an individual plays an essential role in predicting levels of empathy fatigue. Generally, nurses in better health exhibit lower empathy fatigue scores, likely because healthier individuals possess greater physical resilience and vitality (30).

### 4.2 Current situation of empathy fatigue in emergency nurses

The findings of this study reveal a high level of empathy fatigue among emergency nurses, aligning with the research conducted by Meimei et al. (5, 35–37). This condition likely arises from the numerous challenges that emergency nurses encounter, which include dealing with many unpredictable elements, special circumstances, a shortage of medical resources, and the need for high-level technical expertise during treatment. Additionally, they face complex social interactions and must manage communications with various stakeholders, such as doctors, patients, patients' families, colleagues, and superiors. Emergency nurses are required to demonstrate considerable resilience and improve their communication skills, especially for managing stress in interpersonal interactions during emergency care. Consequently, hospital managers should effectively guide the emotions of emergency nurses, offering encouragement and support while fostering their courage, optimism, and positive outlook. It is essential for emergency nurses to adjust their mindset and enhance their ability to cope with stress. They can improve their professional competence and psychological resilience through participation in professional training, acquisition of psychological knowledge, and engagement in personal interests and hobbies. Moreover, developing the interpersonal communication skills of emergency nurses and actively expanding their social support networks are critical for alleviating empathy fatigue. Such factors are pivotal in reducing the impact of this issue. Empathy fatigue among emergency nurses is an urgent problem that needs to be addressed. It is believed that through the concerted efforts of medical institutions, society, and the nurses themselves, empathy fatigue can be effectively mitigated. This would not only promote the physical and mental health of emergency nurses but also ensure the efficient operation of emergency services (38–41). Addressing these challenges is crucial for ensuring that emergency nurses are fully prepared to excel during critical moments and contribute effectively to life-saving efforts.

### 4.3 Status quo of organizational support among emergency nurses

The findings of this study reveal that the average organizational support score for emergency nurses is 41.54 ± 12.35. The scores across different dimensions of support were as follows: instrumental support averaged 3.10 ± 0.85, marginally higher than affective support, which averaged 3.02 ± 0.87. These results indicate that the level of organizational support provided to emergency nurses in hospital settings is moderate, with affective support being slightly lower than instrumental support. This observation is consistent with the results of previous surveys (42–47). There is a clear need for hospitals to improve their support for emergency nurses, especially in terms of emotional support. When emergency nurses genuinely feel valued and recognized by their organization, they are more likely to remain committed and enthusiastic about their roles. The univariate analysis showed that emergency nurses over the age of 41 and those holding higher professional titles reported greater organizational support. This finding aligns with Ma Lifang's research (48–50). Typically, older nurses are more accustomed to the work environment, more at ease with their duties, have a stronger sense of community and personal identity, and thus perceive higher levels of organizational support. Nursing managers can better cater to nurses' needs by organizing departmental exchange meetings, promoting mentorship programs where experienced nurses guide their less experienced peers in both professional and personal matters, and by increasing emotional support for all nursing staff. Moreover, the study found significant differences in perceived organizational support depending on the department and the number of night shifts worked per month, corroborating Jia Zhaonan's findings (51, 52). Since salary can be regarded as a form of organizational support, it follows that emergency nurses with higher monthly incomes perceive greater organizational support. To better support emergency nurses, hospitals should seek additional resources, improve working conditions, foster a positive work environment, reduce psychological burdens, and enhance job satisfaction and overall outlook (53, 54).

### 4.4 Psychological capital status of emergency nurses

The study revealed that the overall psychological capital score for emergency nurses stood at 77.89 ± 10.73 points, with an average score per item of 3.82 ± 1.01. This indicates that the performance was above the median level. Specifically, the scores were distributed as follows: self-efficacy scored 3.90 ± 0.99, hope scored 3.73 ± 1.05, resilience scored 3.82 ± 1.04, and optimism scored 3.81 ± 1.14. Each of these dimensions exceeded the median level. This research aligns with findings from other relevant studies in China, suggesting that China's emergency nurses maintain a relatively high level of psychological capital. Nonetheless, when compared to global benchmarks, there remains potential for further improvement and development (55, 56). Psychological capital is recognized as a distinct psychological resource that contributes positively by fostering optimism, resilience, hope, and self-efficacy in everyday life. It is shaped by the work environment, and prolonged exposure to challenging conditions can reduce psychological capital. According to class state theory, psychological capital is not only measurable and developable but also evolves over a person's lifetime. Elements such as self-confidence, hope, resilience, and optimism not only gradually develop through long-term work and life experiences but also become stable aspects of an individual's personality. Furthermore, these elements can be strengthened through effective interventions or training, thereby enhancing an individual's mental health and improving their ability to cope with challenges in both work and life (57–61).

### 4.5 The mediating role of psychological capital between organizational support and empathy fatigue

The mediation effect test results indicate that psychological capital can act as a partial mediator between organizational support and empathy fatigue among emergency nurses, accounting for 27% of the total effect. These findings reveal that organizational support not only has a direct impact on empathy fatigue but also indirectly influences it through psychological capital (18, 62). Organizational support includes both instrumental and emotional components. Research shows that strong organizational support can effectively and positively predict psychological capital, and higher levels of support enhance the psychological capital of emergency nurses more efficiently (9). Improving nurses' sense of belonging enhances their occupational identity, while bolstering their self-efficacy to manage stress increases their optimism and promotes their career planning abilities. Furthermore, organizational support provides essential assistance and encouragement to emergency nurses when they encounter challenges, thereby enhancing their resilience and enabling them to better manage stress and adversity. Consequently, the boost in emergency nurses' psychological capital further improves their perception and utilization of organizational support, creating a virtuous cycle that enhances the performance and overall development of emergency nurses (23). Moreover, organizational support is vital in mitigating empathy fatigue. The nature of emergency nursing often requires nurses to continuously connect emotionally with others, empathize deeply, and share in their emotions, which can deplete their emotional resources. Emotional support from organizations, such as understanding, care, and recognition, helps nurses feel valued and significant, thus compensating, to some extent, for the depletion of emotional resources (63). Psychological capital is a positive psychological state that encompasses self-efficacy, optimism, hope, and resilience. Nurses endowed with these psychological resources are more resilient and better equipped to handle traumatic work-related events. They also adapt more effectively to high-pressure healthcare environments, thereby alleviating empathic stress and reducing the incidence of empathy fatigue (64). Studies suggest that nurses with low self-efficacy are more vulnerable to negative emotions and are likely to make negative judgments in stressful situations, which can decrease job satisfaction. In contrast, possessing optimism and hope, which are forms of positive cognitive and emotional experiences, enables nurses to positively confront traumatic events, thus diminishing empathy fatigue (65). Resilience, defined as an individual's capacity to respond effectively to adversity or challenging situations, is crucial. Emergency nurses with high resilience can adapt well, face challenges courageously, and recover swiftly from the negative impacts related to patient care (66). Society and healthcare institutions should recognize the importance of these dynamics, offer more organizational support to emergency nurses, and foster their positive psychological capital, such as optimism, resilience, hope, and self-efficacy. Doing so will help reduce the level of empathy fatigue among emergency nurses, thereby achieving the goals of enhancing the quality of emergency nursing services, maintaining the stability of the emergency nursing workforce, and promoting the sustainable development of the emergency nursing field.

## 5 Countermeasures and suggestions

Firstly, emergency nurses should enhance their overall quality, improve their stress resilience, learn emotional regulation, and increase their psychological capital levels to approach emergency work with enthusiasm and commitment. Secondly, nursing managers should tailor their instruction, communication, coordination, and planning to individual needs, fostering a positive work environment and subtly reinforcing the organization's care and attention toward emergency nurses. Finally, alongside providing material support, hospitals should also offer emotional support to emergency nurses and actively work to reduce the phenomenon of “emphasizing medicine over nursing” (67–70), thereby lessening their levels of empathy fatigue.

## 6 Limitations

Firstly, although we controlled for some potential confounders such as gender, number of night shifts, and health status, other confounding variables were not considered, and future studies should include more covariates to minimize interference in effect estimates. Secondly, only emergency nurses from healthcare organizations in the Chengdu-Chongqing region were included, and the results may not be representative due to geographical and cultural constraints. Additionally, this study was not comprehensive enough to assess the psychological state of the participants, despite collecting data based on specific indicators used in the analyses. Future research could provide a more in-depth assessment of psychological factors, which could help enrich our findings.

## Data Availability

The original contributions presented in the study are included in the article/supplementary material, further inquiries can be directed to the corresponding author.
